# Supplementary material for: Tracing Ion Migration in Halide Perovskites with Machine Learned Force Fields
Source: J Phys Chem Lett. 2025 May 15;16(20):5153–9. doi: 10.1021/acs.jpclett.5c01139 (PMC12105036; doi:10.1021/acs.jpclett.5c01139)
Supplement: Supplementary file 1 [file jz5c01139_si_001.pdf]

Supporting Information: Tracing Ion Migration in Halide Perovskites With Machine Learned Force Fields

Viren Tyagi,[†,‡] Mike Pols,[†,‡] Geert Brocks,[†,‡,¶] and Shuxia Tao[*,†,‡]

†*Materials Simulation & Modelling, Department of Applied Physics and Science Education, Eindhoven University of Technology, 5600 MB, Eindhoven, The Netherlands*

‡*Center for Computational Energy Research, Department of Applied Physics and Science Education, Eindhoven University of Technology, 5600 MB, Eindhoven, The Netherlands*

¶*Computational Chemical Physics, Faculty of Science and Technology and MESA+ Institute for Nanotechnology, University of Twente, 7500 AE, Enschede, The Netherlands*

E-mail: s.x.tao@tue.nl

Contents

1 Structure optimization and electronic properties **S3**

2 Charge density analysis **S7**

3 Force field training **S9**

4 Spin-orbit coupling **S13**

5 $CsPbI_3$ phase transition **S16**

6 Force field validation **S18**

7 Production runs and diffusion coefficients **S22**

8 Meyer-Neldel rule **S25**

9 Motion of the bridge iodide atoms **S26**

References **S27**

1 Structure optimization and electronic properties

Density functional theory (DFT) calculations were performed with the Vienna Ab-initio Simulation Package (VASP).[1] The projector-augmented wave (PAW) technique[2] was used to model the electron-ion interaction, with the outermost electrons of I ($5s^2 5p^5$), Cs ($5s^2 5p^6 6s^1$) and Pb ($6s^2 6p^2$) treated as valence electrons, and applying the standard VASP PAW potentials. The electronic interactions were modeled using the PBE-D3-BJ exchange-correlation functional within the generalized gradient approximation (GGA) supplemented with Grimme-type Vanderwaals contributions.[3–5] Energy and force convergence criteria of 10^{-6} eV and 10^{-2} eV/Å were used for structure optimization. The calculations were performed with a $2 \times 2 \times 3$ Monkhorst-Pack k-point grid[6] (in the Brillouin zone of the supercells described below) and a kinetic energy cutoff of 300 eV.

As we have shown in a previous study,[7] PBE-D3-BJ, the functional of choice for this study, gives reliable defect formation energies at a moderate computational cost. In particular, we highlight that the inclusion of Vanderwaals interactions is very important. We use the PBE-D3-BJ functional for all DFT calculations in this paper, including in training the machine-learned force fields (MLFFs). To reduce computational costs, we neglected spin-orbit coupling (SOC) in MLFF training. This has only a small influence on the forces, as shown in Section 4.

We used the optimized orthorhombic $CsPbI_3$ pristine unit cell of the ground state equilibrium structure as a building block. We created single iodide point defects by removing or adding an iodide atom to form a vacancy ${V_I}^0$ or interstitial ${I_I}^0$, respectively, in $2 \times 2 \times 1$ supercells (16 units of $CsPbI_3$) of the optimized pristine structure. To create the different charge states of these defects, we either added or removed one electron from the total number of electrons for systems with neutral defects. Finally, we optimized all these structures using the aforementioned DFT parameters and convergence criteria.

The optimized defect geometries are given in Figure S1. The Pb atoms are more closely bonded to the iodide interstitial in ${I_I}^-$ (Figure S1a) than in ${I_I}^0$ (Figure S1b), whereas in case

I_I^+ the interstitial is in the center of a face of the Pb−I cage (Figure S1c). For an iodide vacancy, the Pb atoms adjacent to the defect are closer to each other in the case of V_I^- (Figure S1d) than for V_I^0 (Figure S1e), and these two atoms were the furthest apart for V_I^+ (Figure S1f). These local structural changes are also quantitatively captured by the distance between the defect adjacent Pb atoms for both iodide interstitial and iodide vacancy, which are given in Table S1. It should be noted that SOC does change the Pb-Pb distance around the I vacancy, without it affecting the potential energy surface much, however, see Section 4.

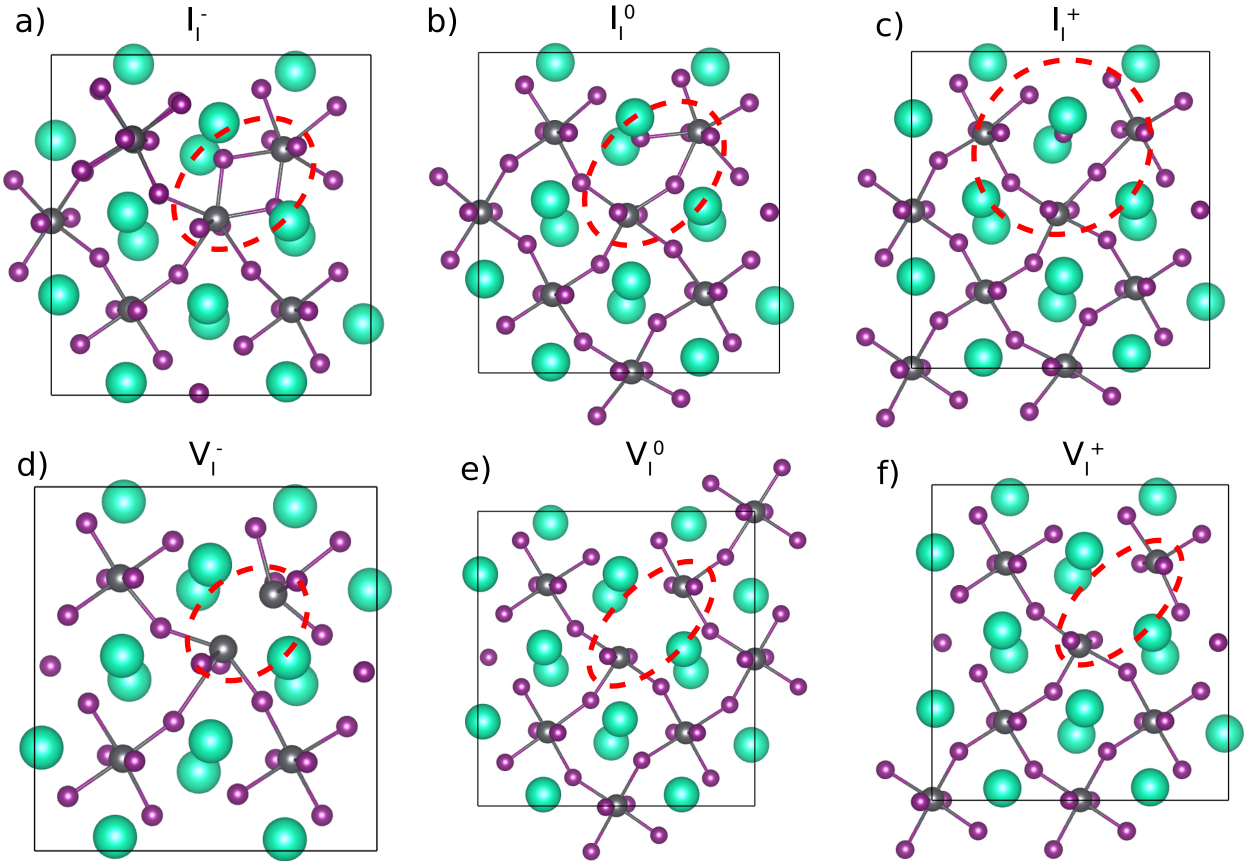

Figure S1: DFT optimized geometries of the different charge states of the iodide interstitial (a-c) and the iodide vacancy (d-f) defects in $CsPbI_3$. The defect environment is circled by dashed red lines.

These optimized structures were used to calculate the band structures shown in Figure S2. For the positive interstitial I_I^+ there is an unoccupied defect level approximately 1

Table S1: Distance between the defect adjacent Pb atoms for the optimized geometries of the different charge states of the iodide interstitial and vacancy defects in $CsPbI_3$.

System	Pb−Pb distance (Å)
I_I^-	5.10
I_I^0	6.22
I_I^+	6.64
V_I^-	3.65
V_I^0	5.84
V_I^+	6.54

eV above the Fermi energy (Figure S2c). The lack of dispersion of this level indicates that the corresponding state is localized, which is confirmed by the charge distributions shown in Section 2. For the neutral state of the iodide interstitial I_I^0, the Fermi level is located on the localized defect state, which is half filled (Figure S2b). Note that the defect level has dropped in energy with respect to the top of the valence band, as compared to the I_I^+ defect level. When a further electron is added to create the negative state I_I^-, the defect level drops further, and coincides with the top of the valence band (Figure S2a), where the Fermi level shifts to above the defect state. Similarly, for the iodide vacancy V_I^-, an localized occupied defect state can be identified in the band gap, with the Fermi level above the defect state (Figure S2d), see Section 2. Removing one electron to create the neutral vacancy V_I^0 raises this defect level to the bottom of the conduction band, and it becomes half filled (Figure S2e). Finally, for the positive state V_I^+ the Fermi level moves to below the defect level (which coincides with the bottom of the conduction band) (Figure S2f). The band structures make physical sense, and we expect the qualitative analysis to remain the same even if SOC and many-body corrections would be included.

In calculations, one fixes the number of electrons, which determines the position of the Fermi level. In experiment, one would vary the (quasi) Fermi levels in the perovskite by changing the operating the device in which the material is embedded. It is clear that by changing the Fermi level across the band gap one can alter the charge state of the defects. In equilibrium, with the Fermi level inside the band gap pinned by an overall charge neutrality

condition,[7] interstitials are negatively charged, I_I^-, and vacancies are positively charged, V_I^+. Sufficiently lowering the (quasi) Fermi level by injecting holes renders the interstitials positively charged, I_I^+, whereas increasing the (quasi) Fermi level by injecting electrons ultimately gives negatively charged vacancies, V_I^-. Varying the concentration of charge carriers, different charge states of the point defects become relevant. The mobility of such defects thus indirectly depend on the concentration of charge carriers.

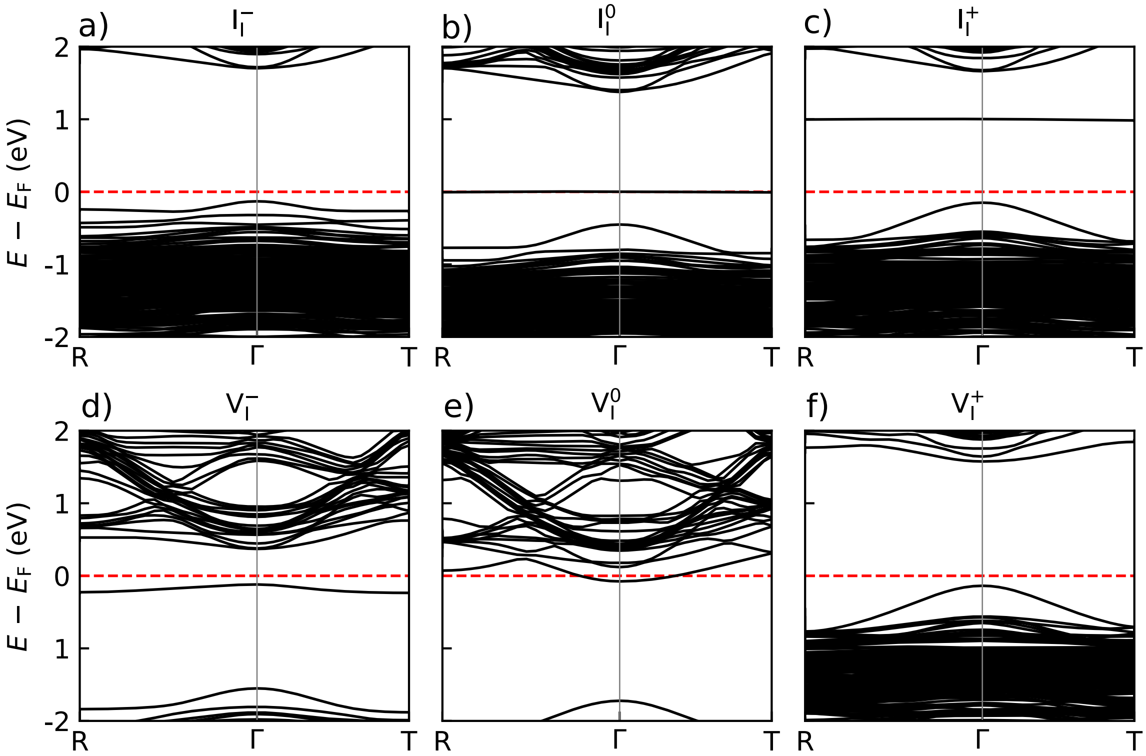

Figure S2: Band structures of defective $CsPbI_3$ systems with different charge states for the (a-c) interstitial and (d-f) vacancy point defects. The Fermi level E_F is taken as the origin, and is represented by dashed lines.

2 Charge density analysis

To check how the charge distribution changes when the charge state of the defects is changed, we compared the charge densities of charged iodide interstitial (${I_I}^-$ and ${I_I}^+$) and iodide vacancy (${V_I}^-$ and ${V_I}^+$) with their neutral counterparts. We started by calculating the charge density of the charged systems using their optimized structures, using these structures we then calculated the charge densities in the neutral case. The charge density difference for all charge systems is given in Figure S3. As evident from the figure, while the charge is delocalized for ${I_I}^-$ (Figure S3a), in case of ${I_I}^+$ it is localized around the iodide interstitial (Figure S3a), validating the character of the defect states observed in the previous section (Figures S2a and c). In case of iodide vacancy, while the charge is delocalized for ${V_I}^+$(Figure S3d), for ${V_I}^-$ it localizes around the iodide vacancy (Figure S3c), again in agreement with the band structures (Figures S2d and f). Similar trends were found by Meggiolaro et al.[8] for the orbital associated with the trapped electron on the ${V_I}^0$ defect in $MASnI_3$.

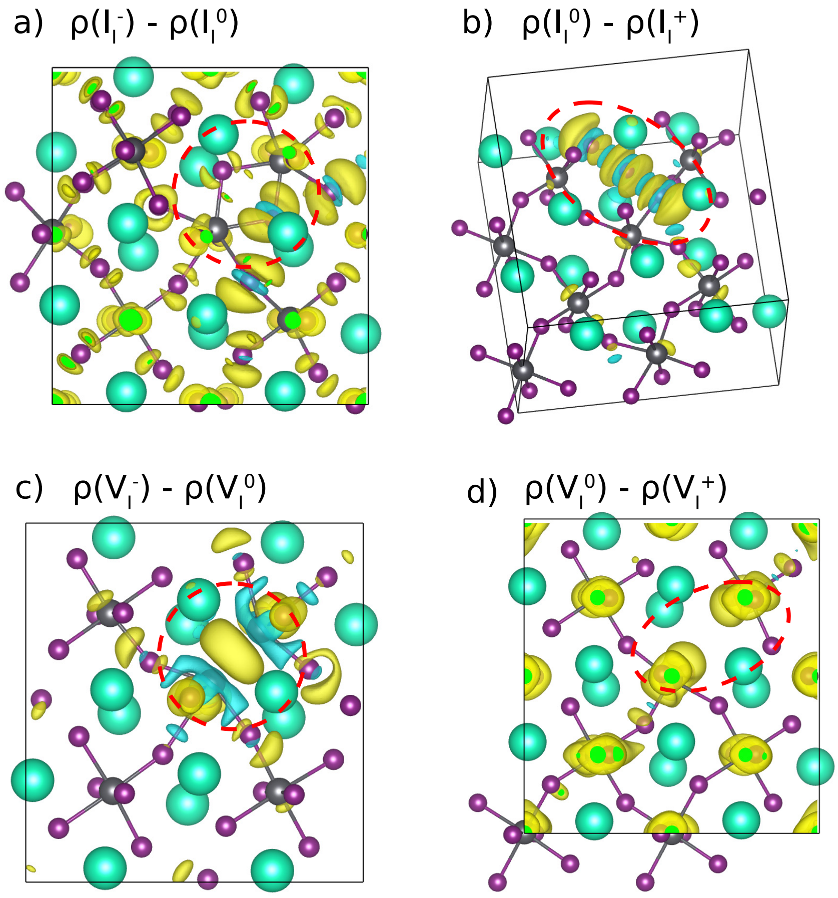

Figure S3: The charge density difference between the charged and neutral iodide interstitials (a,b) and charged and neutral iodide vacancies (c,d). The regions with a negative charge are colored yellow, and the regions with a positive charge are colored blue. The defect environment is circled by dotted red lines.

3 Force field training

Training structures were sampled from three short timescale molecular dynamics (MD) runs performed in VASP.[9] These runs were performed at different temperatures for each system to ensure that force fields could describe diverse atomic environments during the production runs. Temperatures for these runs are given in Table S2. Almost all of these runs were performed on $2 \times 2 \times 2$ supercells (in units of the cubic cell, i.e., 8 units of $CsPbI_3$ in total) for 100 ps with MD time-steps of 2 fs. The only exception was the final training step for V_I^-, for which a $3 \times 3 \times 3$ supercell (27 units of $CsPbI_3$) was used because the dynamics of lead atoms away from the defect environment was not described well with smaller supercells. As a larger supercell was used in this case, the run was only 40 ps long. Furthermore, most of these runs were performed in NpT ensembles at 10^5 Pa pressure. To maintain constant temperature and pressure, Parinello-Rahman dynamics was used.[10,11] The values of the friction coefficients for all atomic and lattice degrees of freedom were set to 3 ps^{-1}. Unless mentioned otherwise, these thermostat and barostat settings were used for all MD runs. For both V_I^- and I_I^- defects, unphysically large volume expansions were observed at high temperatures ($\geq$ 700K), possibly due to the excess electronic charge in relaticely small training structures. Consequently, the volume was kept constant during these runs, while the shape of the cell was allowed to change.

Table S2: Temperatures at which training was performed for each defect system. Here * corresponds to the runs for which the cell volume was kept constant, whereas # denotes the run performed using a $3 \times 3 \times 3$ supercell.

System	Step 1	Step 2	Step 3
I_I^-	750 K*	600 K	700 K*
I_I^0	700 K	750 K	600 K
I_I^+	700 K	500 K	600 K
V_I^-	750 K*	700 K*	700 K*#
V_I^0	750 K	700 K	600 K
V_I^+	750 K	750 K	650 K

During these training runs, the frames were evaluated using DFT calculations whenever

the estimation error in forces was higher than a threshold value. The DFT parameters were set as described in Section 1, except for the force convergence criterion, which was tightened to 10^{-5} eV/Å. Only for V_I^- the convergence criteria turned out to be too strict, and were relaxed to 10^{-4} eV and 10^{-3} eV/Å for the total energy and forces respectively. The calculations were performed with a $2 \times 2 \times 2$ Γ-centered k-point grid and a kinetic energy cutoff of 300 eV.

To describe the local chemical environments, a combination of two descriptors similar to the smooth overlap of atomic orbitals (SOAP) descriptor was used.[12,13] The first of these was a simple radial distribution function that can also be regarded as a two-body descriptor, for which a cutoff $\rho_i^{(2)}$ of 7 Å was used. The second descriptor is purely angular containing no two-body components, for which a cutoff $\rho_i^{(3)}$ of 6 Å was used. The atomic positions were expanded using Gaussian distributions of width 0.5 Å. The radial descriptor was expanded using 8 radial basis functions, whereas the angular descriptor was expanded using 6 radial basis functions and spherical harmonics with a maximum angular momentum quantum number L_{max} of 6. As the structures were sampled, a set of local reference configurations was constructed. The maximum size of this set was 4000 for V_I^-, 3500 for I_I^-,I_I^0, and V_I^+, and 3000 for V_I^0, and I_I^+. The local potential energy of an atom in a structure from the training set was expressed as a linear combination of Gaussian kernels that measure the similarity between local reference configuration from the training set and the basis set.[9] A polynomial power of 4 was used for these kernels, and the weight of the radial descriptor in these kernels was set to 0.1, setting the weight of the angular descriptor to 0.9.

The forces and energies predicted by the current force field can be compared with forces and energies calculated using DFT during training. The cumulative root mean squared difference between these forces over training time is given in Figure S4. These errors plateau over time for all systems, signifying the force fields learn the material dynamics of the defective perovskite systems. The root mean squared errors in energies are given in Table S3. As the energies of the training structures are of the order of eV/atom these values

indicate that the force fields are accurately learning the energies during training. Finally, all the force fields were refitted onto faster descriptors without any error estimate to speed up their evaluations for use in production runs. The number of structures in the training sets (N_{DFT}) and the number of local reference configurations for the different elements in the MLFFs (N_{basis}) are given in Table S4

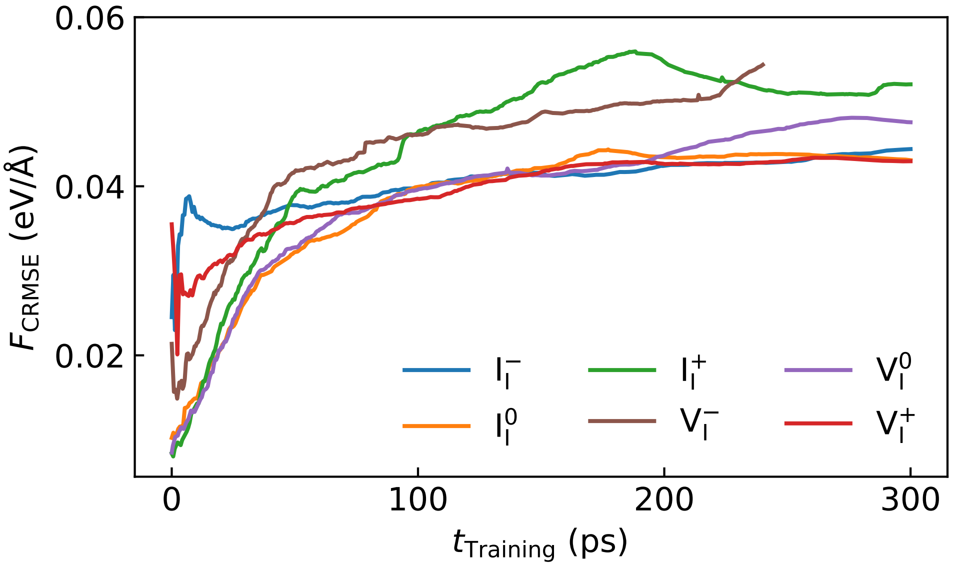

Figure S4: Cumulative root mean squared error (CRMSE) in forces calculated by the force field compared to DFT calculated forces during training.

Table S3: Root mean squared error (RMSE) in energies calculated by the force fields compared to DFT calculated energies during training.

System	E_{RMSE} (meV/atom)
$\mathrm{I_I^-}$	1.38
$\mathrm{I_I^0}$	1.42
$\mathrm{I_I^+}$	2.35
$\mathrm{V_I^-}$	3.68
$\mathrm{I_I^-}$	1.76
$\mathrm{I_I^-}$	1.25

Table S4: Size of the training set and number of local reference configurations in the MLFFs for each defect system.

System	$N_{\mathrm{DFT}}(-)$	$N_{\mathrm{basis}}(-)$		
		Cs	Pb	I
$\mathrm{I_I}^-$	2217	1752	1457	3500
$\mathrm{I_I}^0$	2008	1444	1184	3500
$\mathrm{I_I}^+$	2176	1371	1006	3000
$\mathrm{V_I}^-$	2371	1819	2536	4000
$\mathrm{V_I}^0$	2715	1821	1990	3000
$\mathrm{V_I}^+$	1827	1600	1291	3500

4 Spin-orbit coupling

To test the influence of spin-orbit coupling (SOC), we first optimized all the defect geometries including SOC, using the same DFT parameters as in Section 1. The distance between the two Pb atoms closest to the defect in Table S5. Upon comparing these results with defect geometries optimized without SOC (Table S1), we note that for iodide interstitials, SOC has a minimal influence on the defect geometry. In contrast, for iodide vacancies, the distance between the defect adjacent Pb atoms increases upon the inclusion of SOC. This is especially true for V_I^-, where this distance increases from 3.65 Å to 6.13 Å, which is in agreement with the results reported by Meggiolaro et al.[14]

Besides defect geometries being affected, it is more interesting to observe the effects of SOC on the potential surface and the forces, as the latter determine the dynamics of the system in the MD runs. We sampled 20 structures from the highest temperature training run for each system and compared the forces calculated using DFT with and without SOC. The forces categorized to atomic species are given in Figure S5, and the mean absolute errors (MAE) of these comparisons are given in Table S6. We note that forces calculated using DFT without SOC are in very close agreement with forces calculated with SOC, as is evident by having $R^2 = 0.99$ and MAE ≤ 22.66 meV/Å for all defect systems. As the forces acting on the atoms are of the order of eV/Å, these errors are small.

From these comparisons we conclude that while the inclusion of SOC affects the optimized defect geometry of iodide vacancies, especially that of V_I^-, it has no significant influence on forces, and hence, we do not include it during model training to limit the computational cost.

Table S5: Distance between the defect adjacent Pb atoms for the optimized geometries of the different charge states of the iodide interstitial and vacancy defects in $CsPbI_3$ optimized with spin-orbit coupling.

System	Pb−Pb distance (Å)
I_I^-	5.10
I_I^0	6.25
I_I^+	6.64
V_I^-	6.13
V_I^0	6.40
V_I^+	6.53

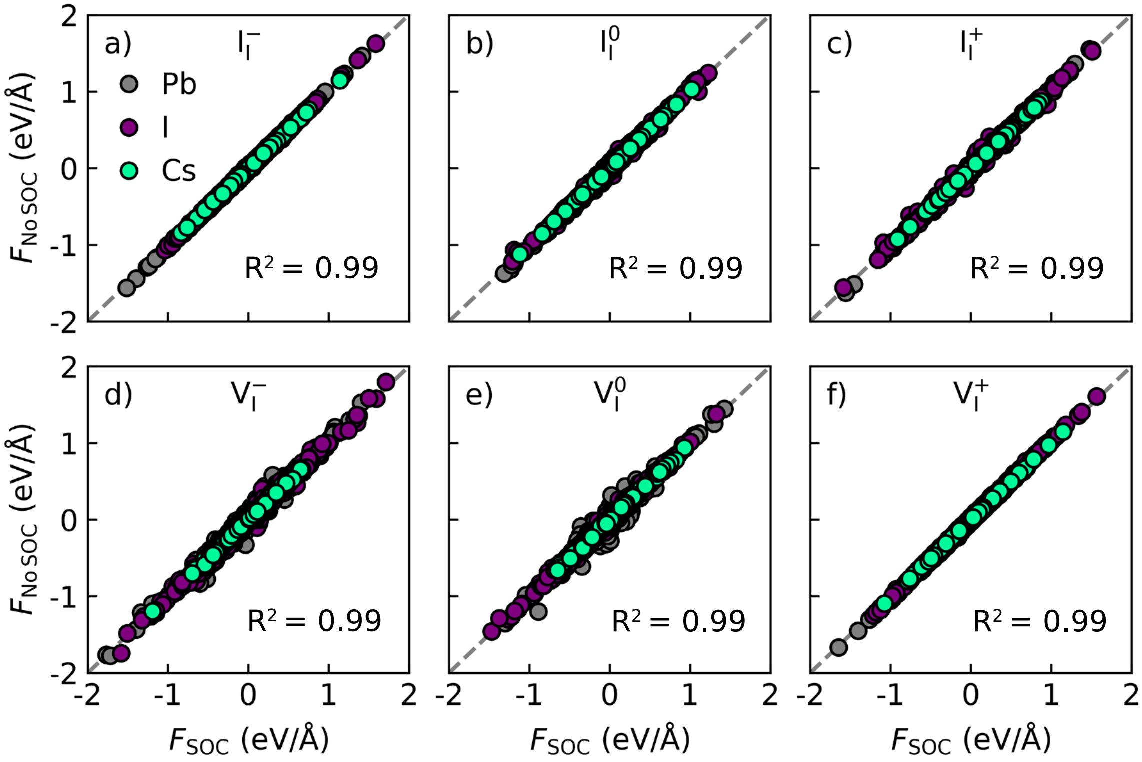

Figure S5: Comparison between forces calculated using DFT with spin-orbit coupling (SOC) and forces calculated using DFT without spin-orbit coupling (No SOC) on training structures of all defect systems, along with their corresponding R^2 values.

Table S6: Mean absolute error (MAE) between forces calculated using DFT with spin-orbit coupling and forces calculated using DFT without spin-orbit coupling on training structures of all defect systems.

System	MAE (meV/Å)
${I_I}^-$	11.08
${I_I}^0$	14.31
${I_I}^+$	13.91
${I_I}^-$	22.66
${I_I}^-$	20.94
${V_I}^+$	11.90

5 $CsPbI_3$ phase transition

To avoid the influence of volume expansion on diffusion, all equilibration and production runs were performed at constant volume. The cell volumes at different temperatures were deduced from constant temperature MD runs used to study phase transitions in $CsPbI_3$. The MLFF model used for these runs was trained on $2 \times 2 \times 2$ (8 units of $CsPbI_3$) pseudo-cubic cells in all three perovskite phases of $CsPbI_3$. We started the training in the cubic phase at 700 K, followed by the tetragonal phase at 510 K, and finally in the orthorhombic phase at 325 K and 150 K. All these runs were performed at 10^5 Pa pressure for 100 ps with MD time-steps of 5 fs. For the DFT calculations we used the same functional, PAW potentials and kinetic energy cutoff as in Section 1, force and energy convergence criteria of 10^{-4} eV and 10^{-3} eV/Å, and a $2 \times 2 \times 2$ Γ-centered k-point grid. To construct the MLFF, cutoff radii of 6 Å and 5 Å were used for the radial and the angular descriptors, respectively. The atomic positions were expanded using Gaussian distributions of width 0.5 Å. The radial descriptor was expanded using 6 radial basis functions, and the angular descriptor was expanded using 6 radial basis functions and spherical harmonics with a maximum angular momentum quantum number 6. Gaussian kernels of polynomial power 4 were used, where the radial and angular descriptors have weights 0.7 and 0.3, respectively.

The accuracy of this model was validated by performing a heating MD run from 100 K to 700 K using a $3 \times 3 \times 2$ (72 units of $CsPbI_3$) orthorhombic supercell. The window averaged pseudo-cubic lattice vectors from this run are given in Figure S6, and the phase transition temperatures are in close agreement with experiments.[15]

Using this force field, we performed 100 ps long constant temperature MD runs in the NpT ensemble at various temperatures between 500 K and 600 K. These runs were performed using $6 \times 6 \times 6$ (216 units of $CsPbI_3$) cubic supercells, and the cube root of the final volume was taken as the lattice constant.

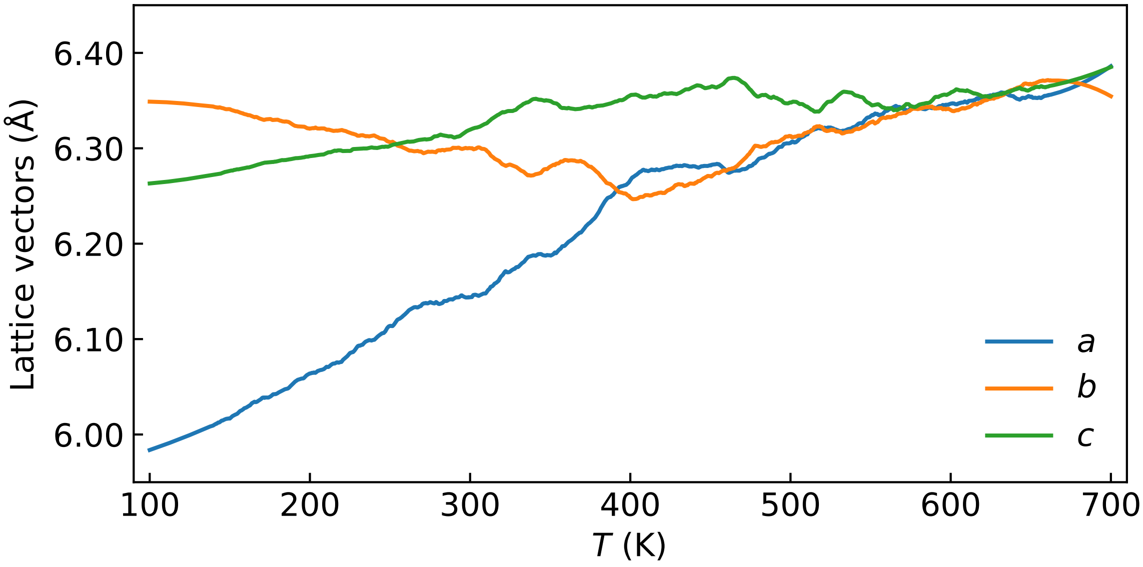

Figure S6: Pseudo-cubic unit cell lattice vectors of $CsPbI_3$ as function of temperature in the heating MD run.

6 Force field validation

To test the accuracy of our MLFFs, we start by comparing the energies predicted by the force fields with those calculated using DFT for migration paths as they would enter transition state theory (TST). These paths were constructed using climbing image nudged elastic band (CI-NEB) calculations[16] on $2 \times 2 \times 1$ orthorhombic supercells (16 units of $CsPbI_3$) with one point defect. Five intermediate images, connected by springs with spring constant $5\,\mathrm{eV/Å^2}$, were optimized, using a cut-off energy of 500 eV and a single k-point (Γ-point), where for ${I_I}^0$ and ${I_I}^-$ only three intermediate images were optimized. The computed energy barriers for defect migration are shown in Table S7. The differences between the barriers calculated with DFT and with the MLFFs generally are on the scale of 0.1 eV or better, with the exception of ${V_I}^0$, where the difference is on the scale of 0.2 eV.

Table S7: Defect migration barriers (E_b) calculated using DFT/TST/CI-NEB migration paths.

System	E_b^{DFT} (eV)	E_b^{MLFF} (eV)
${I_I}^-$	0.36	0.27
${I_I}^0$	0.14	0.16
${I_I}^+$	0.49	0.59
${V_I}^-$	1.00	0.86
${V_I}^0$	0.49	0.71
${V_I}^+$	0.44	0.51

To test the accuracy of the MLFFs during the MD production runs, we sampled 20 structures from 0.5 ns MD runs at 600 K performed using $6 \times 6 \times 6$ supercells (216 units of $CsPbI_3$) with one iodide point defect, and compared forces calculated using DFT with forces calculated using the force fields for these structures. We compared forces acting on all atoms and forces acting on the atoms close to the defect to check the accuracy with which the force fields could describe the defect environments. The process used to identify the defect environments is illustrated in Figure S7. For all vacancies (Figure S7a), and ${I_I}^-$ and ${I_I}^0$ (Figure S7b) interstitials, the defect environments were identified by counting the number of iodide atoms coordinated to each lead atom within a 4.5 Å radius. For the defect adjacent

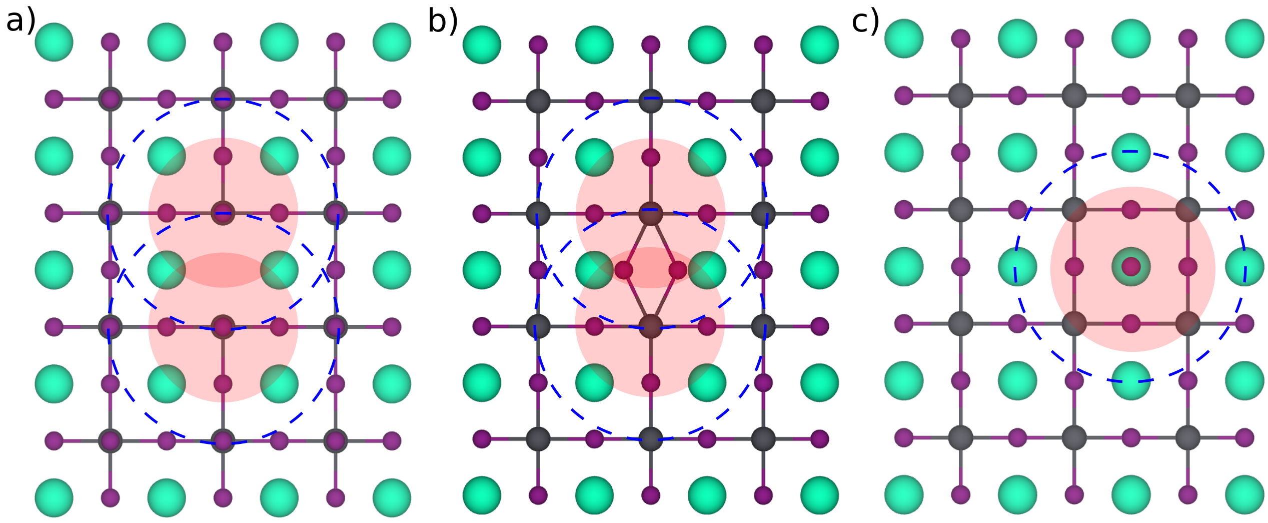

Figure S7: Illustration of how atoms close to the defect environment were identified during the validation runs for a) V_I^-, V_I^0, and V_I^+, b) I_I^- and I_I^0, and c) I_I^+. Here the solid red circles indicate the area used for defect environment identification, and the dashed blue circles indicate the area used for selecting defect environment atoms.

lead atoms, this number was 7 for iodide interstitials and 5 for iodide vacancies. Due to the unique defect geometry of I_I^+, in which the iodide interstitial exists in the center of the Pb−I cage (Figure S7c), we counted the number of lead atoms coordinated to each iodide within a 5 Å radius, for the interstitial this number was 4. Once the defect atoms were identified, atoms within a 7 Å radius of these atoms were chosen as the defect environment atoms. The atomic neighbors were identified using the atomistic simulation environment (ASE) Python package.[17]

The forces categorized according to the atomic species acting on all atoms, and the forces acting on atoms close to the defect environment calculated using the force fields and DFT are given in Figure S8 ,and Figure S9 respectively. The mean absolute errors (MAE) between forces calculated using force fields and forces calculated using DFT for all defect systems are given in Table S8. From these comparisons, we note that all models are highly accurate in calculating forces on all atoms, and transferable to larger systems evident by $R^2 > 0.94$ (Figure S8)and $\mathrm{MAE_{all}} \leq 54.83\,\mathrm{meV/Å}$ (Table S8) for all systems. Further, atoms close to the defect environment were also described well with the lowest R^2 being 0.93 (Figure S9)

and highest MAE_{defect} being 61.89 meV/Å (Table S8) for I_I^+.

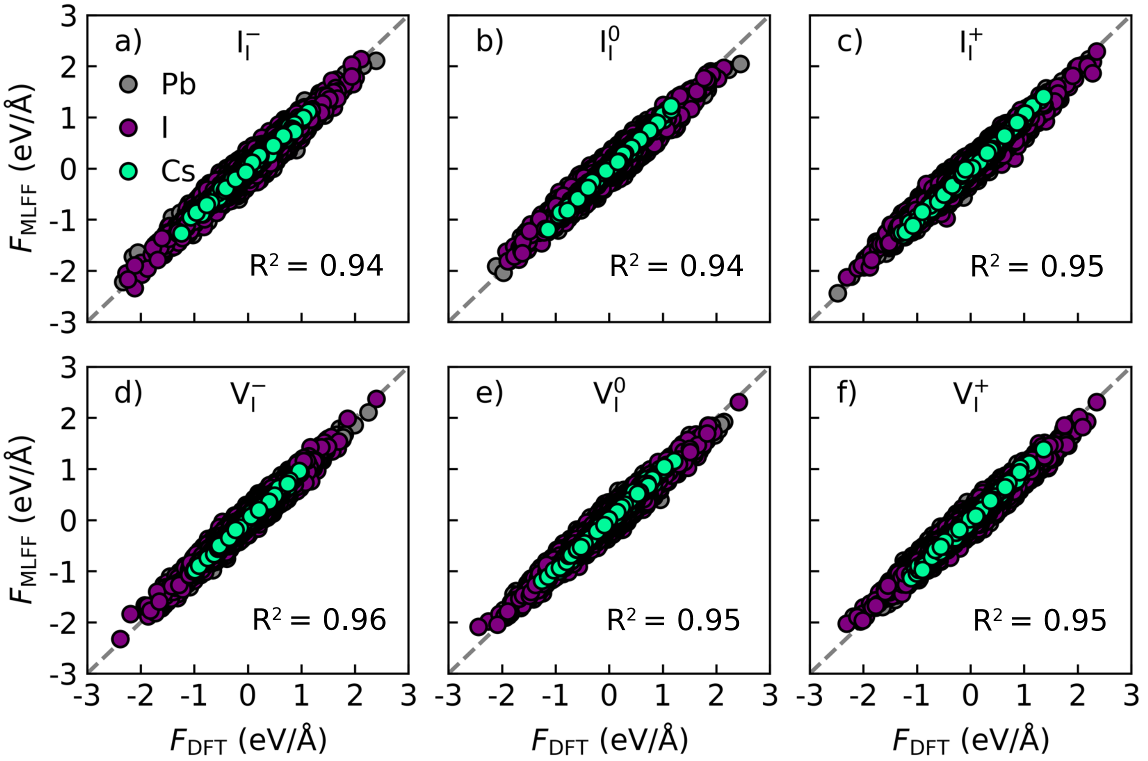

Figure S8: Comparison between forces acting on all atoms calculated by the force fields with forces calculated using DFT for 6 × 6 × 6 supercells of all defect systems, along with their corresponding R^2 values.

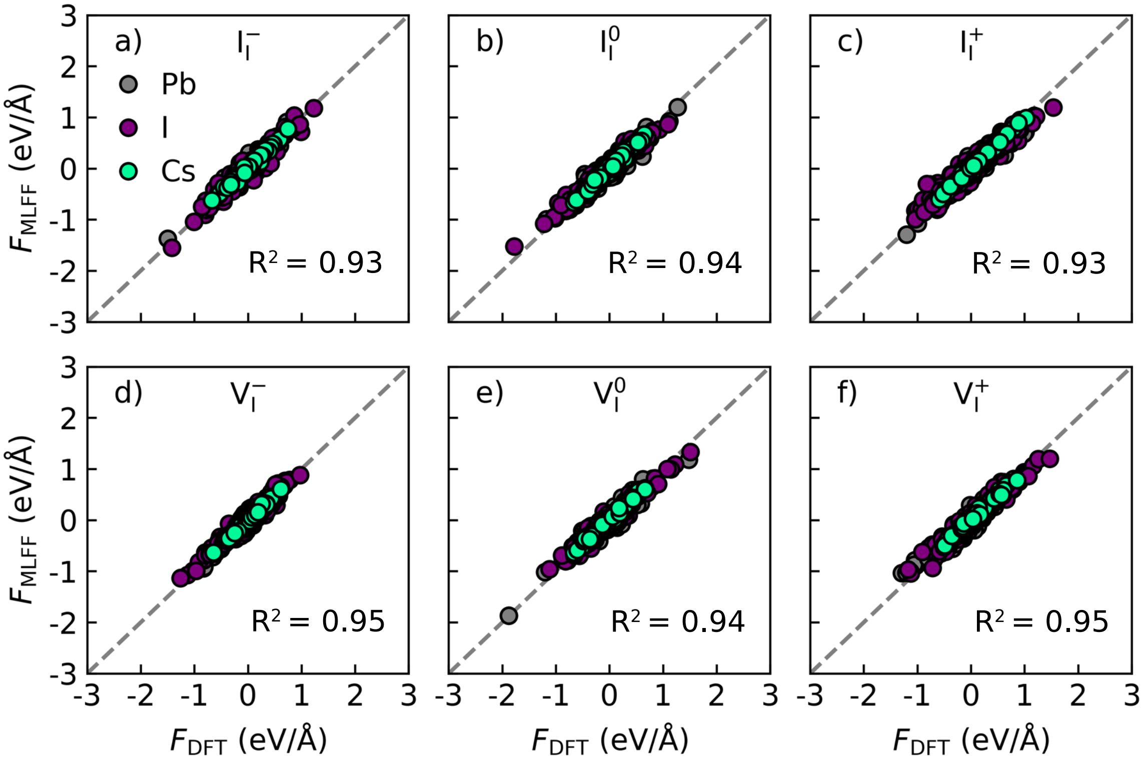

Figure S9: Comparison between forces acting on atoms close to the defect environment calculated by the force fields with forces calculated using DFT for $6 \times 6 \times 6$ supercells of all defect systems, along with their corresponding R^2 values.

Table S8: Mean absolute error (MAE) between forces on all atoms and forces on atoms close to the defect calculated using the force fields and those calculated using DFT for $6 \times 6 \times 6$ supercells of all defect systems.

System	MAE_{all} (meV/Å)	MAE_{defect} (meV/Å)
I_I^-	54.12	52.18
I_I^0	53.85	52.45
I_I^+	54.83	61.89
V_I^-	44.30	47.33
V_I^0	50.97	51.33
V_I^+	52.33	53.44

7 Production runs and diffusion coefficients

Long timescale MD runs were performed at different temperatures using $6 \times 6 \times 6$ cubic supercells (216 units of $CsPbI_3$) for all defect systems using the lattice constants given in Table S9. The latter were obtained as described in Section 5.

Table S9: Lattice constants of $CsPbI_3$ unit cell at different temperatures in cubic phase.

Temperature (K)	Lattice constants (Å)
500	6.324
525	6.330
550	6.336
575	6.343
600	6.349

The structures were first equilibrated at their target temperature for 100 ps with timesteps of 2 fs in an *NVT* ensemble. The atomic positions and velocities of the final frames from the equilibration runs were used as the starting point for 2 ns long production runs. To avoid the influence of a thermostat on diffusion paths,[18] the production runs were performed in the *NVE* ensemble. To ensure the proper sampling of diffusion coefficients, we performed at least 5 production runs at each temperature. A few more runs were performed at low temperatures (500 K and 525 K) for ${I_I}^-$, ${I_I}^+$, and ${V_I}^0$, as the variation in the calculated values of diffusion coefficients was high due to limited defect migration at these lower temperatures for these systems. The number of production runs performed at each temperature for all defect systems is given in Table S10. The average temperatures during the production run for each equilibration temperature, along with fluctuations in temperature are given in Figure S10.

To quantify the migration behavior of the defects, the mean squared displacement (MSD) was plotted over time for each atomic species using the MDAnalysis Python library,[19] where one such plot is given in Figure S11. The MSD was calculated using

$$\mathrm{MSD}(r_d) = \left\langle \frac{1}{N} \sum_{i=1}^{N} |r_d - r_d(t_0)|^2 \right\rangle_{t_0}, \tag{S1}$$

Table S10: Number of production runs performed at each temperature for all defect systems.

System	500 K	525 K	550 K	575 K	600 K
I_I^-	6	6	5	5	5
I_I^0	5	5	5	5	5
I_I^+	10	7	5	5	5
V_I^-	5	5	5	5	5
V_I^0	7	5	5	5	5
V_I^+	5	5	5	5	5

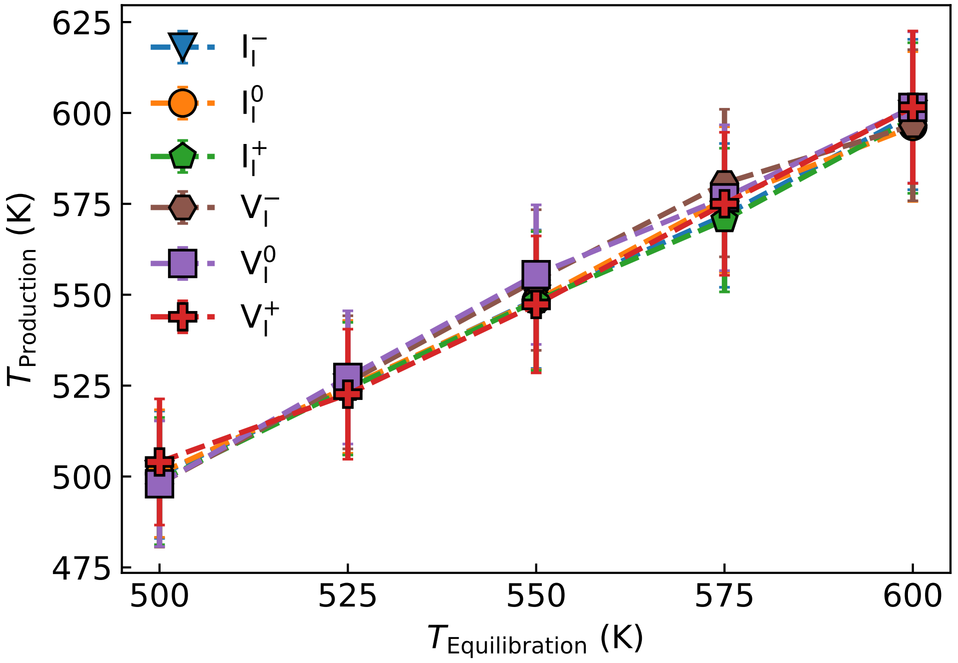

Figure S10: Average temperature during the production runs as a function of equilibration temperature for each defect system. The error bars represent fluctuations in temperatures in the 95% confidence interval.

where N is the number of atoms of a particular atomic species, and r_d are their coordinates in d dimensions (3 for our systems). The Diffusion coefficient D was calculated using

$$D = \frac{N}{2d} \lim_{t \to \infty} \frac{d}{dt} \mathrm{MSD}(r_d), \tag{S2}$$

which is proportional to the slope of the MSD curve, where the factor N ensures that the diffusion coefficient is defect concentration independent.

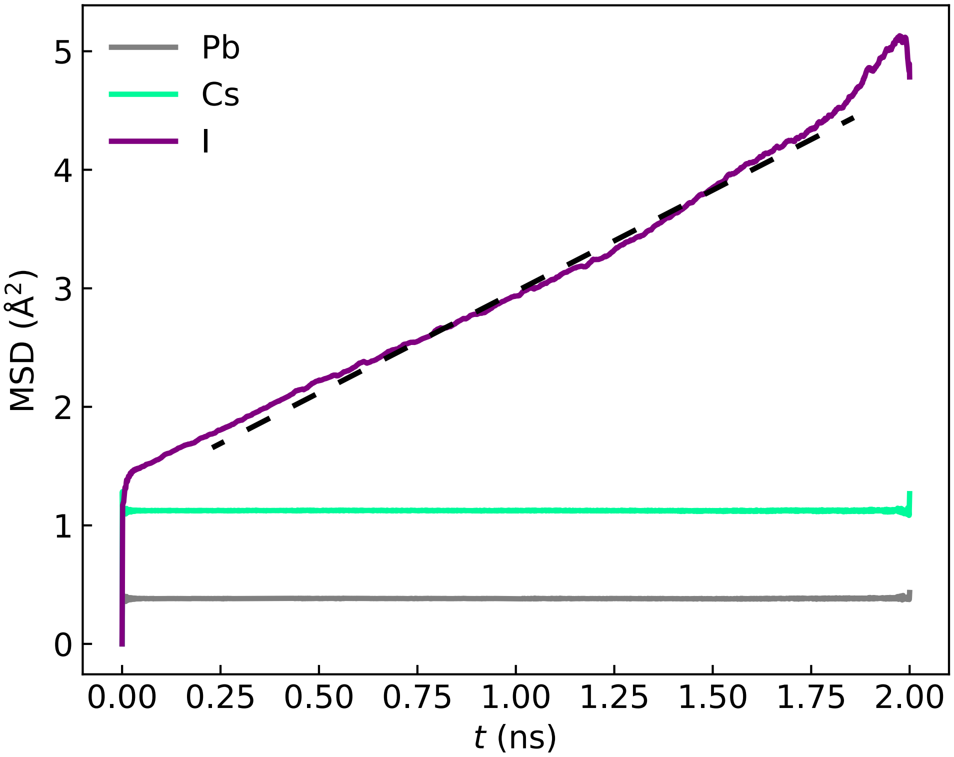

Figure S11: Mean squared displacement (MSD) curves decomposed to the atomic species over simulation time for I_I^0 at 550 K, with the dashed black line taken as the slope of the I MSD curve.

8 Meyer-Neldel rule

The Meyer-Nelder rule[20] states that activation energies (E_a) and pre-exponential factors (D_0) are related through an exponential function. It starts from writing the pre-exponential factor of a reaction rate (which is a diffusion rate in this case) as

$$D_0 = A \exp\left(\frac{S_a}{k_B}\right), \tag{S3}$$

with A a constant, S_a the entropy change at the reaction barrier, and k_B the Boltzmann constant. The Meyer-Nelder rule then states that there is a linear relation between E_a and S_a such that if E_a increases, S_a also increases. One then obtains a linear relation between E_a and $\ln D_0$. This is indeed approximately the case, as is illustrated in Figure S12.

Note that the two factors have an opposite effect on the diffusion rate, i.e., increasing E_a implies that defect migration becomes less probable, whereas at the same time it becomes more frequent because the value of D_0 increases. Hence, the migration rates of all mobile defects lie in a much narrower range than would be expected on the basis of the activation energy alone.

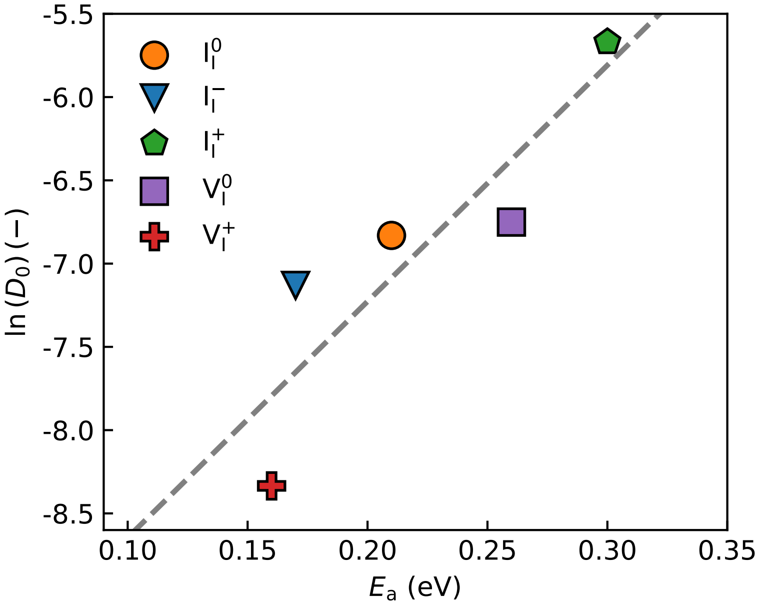

Figure S12: Pre-exponential factors (D_0) versus the activation energies (E_a).

9 Motion of the bridge iodide atoms

The iodide atoms forming the Pb−II−Pb bridge fluctuate less around their mean position in the I_I^- charge state, as compared to the I_I^0 charge state. Taking the lack of fluctuation as a sign of increased bond strength, which is validated comparing their optimized geometries (Figure S1 and Table S1) then it comes as no surprise that I_I^- has a lower migration rate than I_I^0. We calculated the distance between these iodide atoms during the MD runs at different temperatures. These values are given in Figure S13. Here all values smaller than 1.5 Å are excluded as the iodide interstitial was not in the Pb−II−Pb bridge configuration in these frames. As evident from the figure, there is a smaller variance around the mean value of this distance for I_I^- than for I_I^0 at all temperatures, whereas their mean values are very similar.

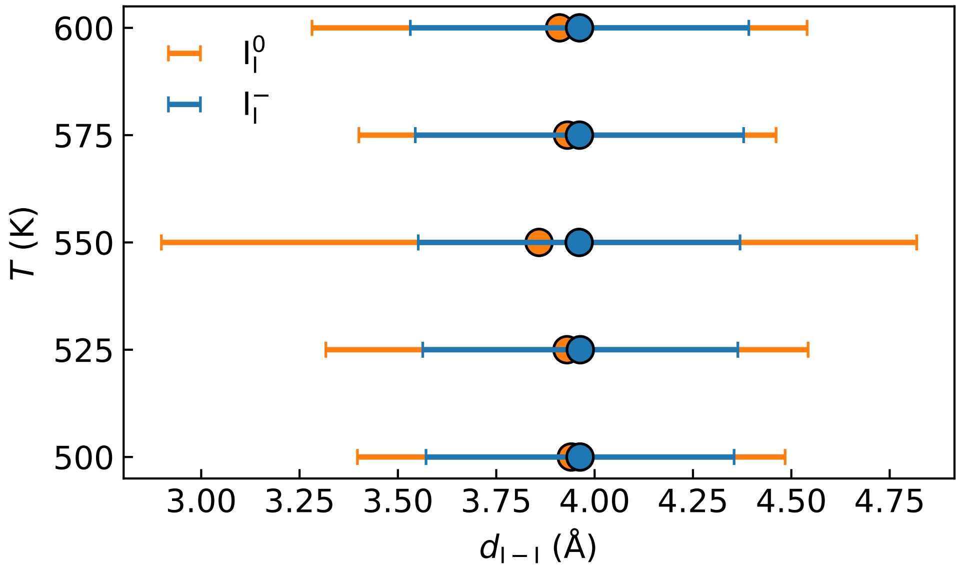

Figure S13: Distance between the iodide atoms forming the Pb−I_I−Pb bridge during the MD runs performed at different temperatures for I_I^- and I_I^0. The circles indicate the mean values.

References

(1) Kresse, G.; Furthmüller, J. Efficient iterative schemes for ab initio total-energy calculations using a plane-wave basis set. *Phys. Rev. B* **1996**, *54*, 11169–11186.

(2) Kresse, G.; Joubert, D. From ultrasoft pseudopotentials to the projector augmented-wave method. *Phys. Rev. B* **1999**, *59*, 1758–1775.

(3) Perdew, J. P.; Burke, K.; Ernzerhof, M. Generalized Gradient Approximation Made Simple. *Phys. Rev. Lett.* **1996**, *77*, 3865–3868.

(4) Grimme, S.; Antony, J.; Ehrlich, S.; Krieg, H. A consistent and accurate ab initio parametrization of density functional dispersion correction (DFT-D) for the 94 elements H-Pu. *J. Chem. Phys.* **2010**, *132*, 154104.

(5) Grimme, S.; Ehrlich, S.; Goerigk, L. Effect of the damping function in dispersion corrected density functional theory. *J. Comput. Chem.* **2011**, *32*, 1456–1465.

(6) Monkhorst, H. J.; Pack, J. D. Special points for Brillouin-zone integrations. *Phys. Rev. B* **1976**, *13*, 5188–5192.

(7) Xue, H.; Brocks, G.; Tao, S. Intrinsic defects in primary halide perovskites: A first-principles study of the thermodynamic trends. *Phys. Rev. Mater.* **2022**, *6*, 055402.

(8) Meggiolaro, D.; Ricciarelli, D.; Alasmari, A. A.; Alasmary, F. A. S.; De Angelis, F. Tin versus Lead Redox Chemistry Modulates Charge Trapping and Self-Doping in Tin/Lead Iodide Perovskites. *The Journal of Physical Chemistry Letters* **2020**, *11*, 3546–3556.

(9) Jinnouchi, R.; Karsai, F.; Kresse, G. On-the-fly machine learning force field generation: Application to melting points. *Phys. Rev. B* **2019**, *100*, 014105.

(10) Parrinello, M.; Rahman, A. Crystal Structure and Pair Potentials: A Molecular-Dynamics Study. *Phys. Rev. Lett.* **1980**, *45*, 1196–1199.

(11) Parrinello, M.; Rahman, A. Polymorphic transitions in single crystals: A new molecular dynamics method. *J. Appl. Phys.* **1981**, *52*, 7182–7190.

(12) Bartók, A. P.; Kondor, R.; Csányi, G. On representing chemical environments. *Phys. Rev. B* **2013**, *87*, 184115.

(13) Jinnouchi, R.; Karsai, F.; Verdi, C.; Asahi, R.; Kresse, G. Descriptors representing two- and three-body atomic distributions and their effects on the accuracy of machine-learned inter-atomic potentials. *J. Chem. Phys.* **2020**, *152*, 234102.

(14) Meggiolaro, D.; De Angelis, F. First-Principles Modeling of Defects in Lead Halide Perovskites: Best Practices and Open Issues. *ACS Energy Letters* **2018**, *3*, 2206–2222.

(15) Marronnier, A.; Roma, G.; Boyer-Richard, S.; Pedesseau, L.; Jancu, J.-M.; Bonnassieux, Y.; Katan, C.; Stoumpos, C. C.; Kanatzidis, M. G.; Even, J. Anharmonicity and Disorder in the Black Phases of Cesium Lead Iodide Used for Stable Inorganic Perovskite Solar Cells. *ACS Nano* **2018**, *12*, 3477–3486.

(16) Henkelman, G.; Uberuaga, B. P.; Jónsson, H. A climbing image nudged elastic band method for finding saddle points and minimum energy paths. *J. Chem. Phys.* **2000**, *113*, 9901–9904.

(17) Larsen, A. H.; Mortensen, J. J.; Blomqvist, J.; Castelli, I. E.; Christensen, R.; Dułak, M.; Friis, J.; Groves, M. N.; Hammer, B.; Hargus, C. et al. The atomic simulation environment—a Python library for working with atoms. *J. Phys. Condens. Matter* **2017**, *29*, 273002.

(18) Basconi, J. E.; Shirts, M. R. Effects of Temperature Control Algorithms on Transport Properties and Kinetics in Molecular Dynamics Simulations. *J. Chem. Theory Comput.* **2013**, *9*, 2887–2899.

(19) Michaud-Agrawal, N.; Denning, E. J.; Woolf, T. B.; Beckstein, O. MDAnalysis: A toolkit for the analysis of molecular dynamics simulations. *J. Comput. Chem.* **2011**, *32*, 2319–2327.

(20) Meyer, W. v.; Neldel, H. Relation between the energy constant and the quantity constant in the conductivity–temperature formula of oxide semiconductors. *Z. tech. Phys* **1937**, *18*, 588–593.
